# Supplementary material for: On the general relationship between plant height and aboveground biomass of vegetation stands in contrasted ecosystems
Source: PLoS One. 2021 May 26;16(5):e0252080. doi: 10.1371/journal.pone.0252080 (PMC8153471; doi:10.1371/journal.pone.0252080)
Supplement: S1 Fig — Box hinges represent first and third quartiles (25th and 75th quantiles). 95th quantiles are 1.38, 1.41, 1.40 kg m-3 for grasslands, prairies and forests, respectively. (DOCX) [file pone.0252080.s001.docx]

**
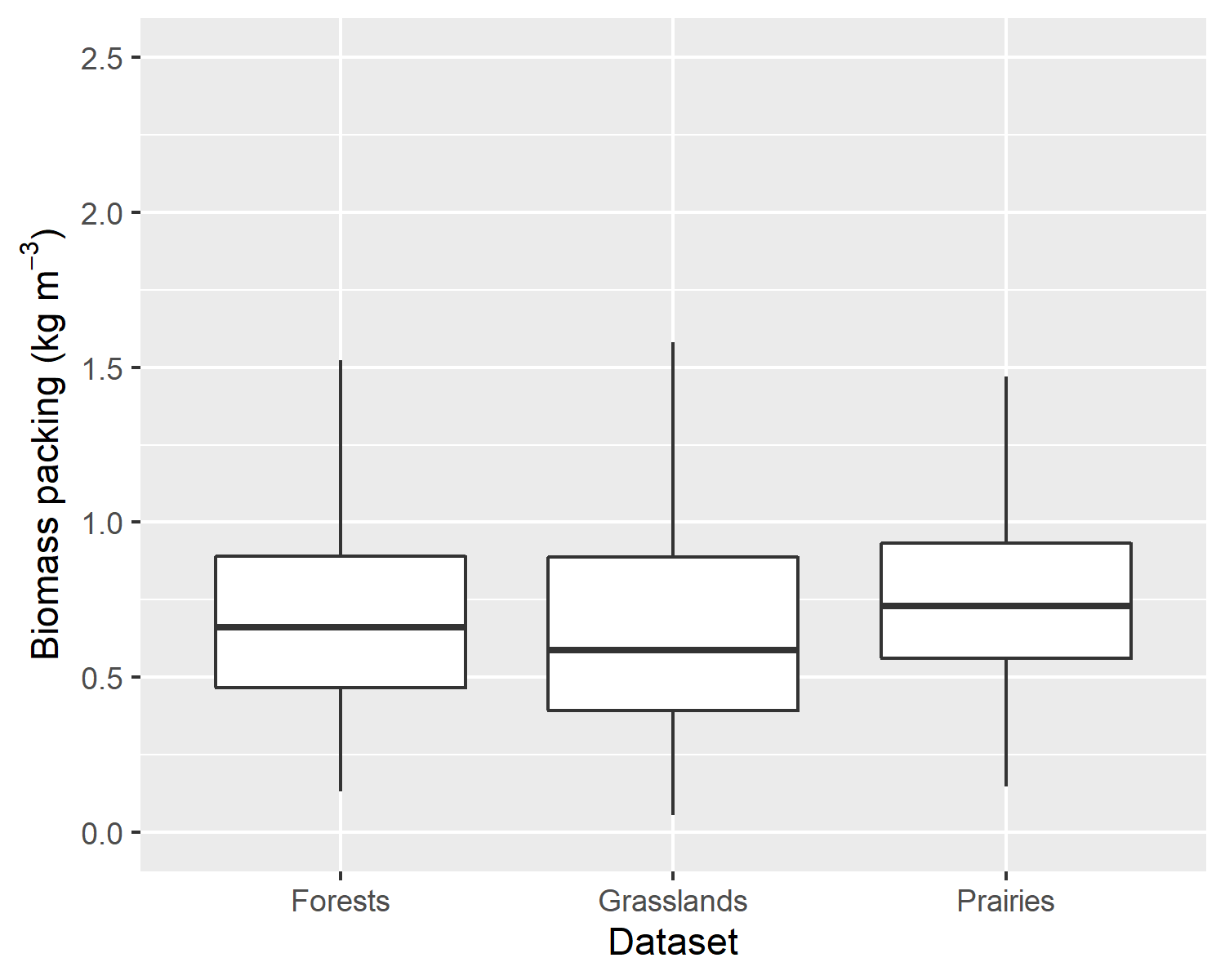
**

**S1 Fig.** Biomass packing distribution of vegetation stands in three ecosystems: Canadian forests (National Forest Inventory), Central Germany managed grasslands (Jena Experiment) and Western US prairies (Cedar Creek Experiment). Box hinges represent first and third quartiles (25th and 75th quantiles). 95th quantiles are 1.38, 1.41, 1.40 kg m^-3^ for grasslands, prairies and forests, respectively.
