# Supplementary material for: On the general relationship between plant height and aboveground biomass of vegetation stands in contrasted ecosystems
Source: PLoS One. 2021 May 26;16(5):e0252080. doi: 10.1371/journal.pone.0252080 (PMC8153471; doi:10.1371/journal.pone.0252080)
Supplement: S2 Fig — The line is the fit of a power function of the form IAB = ·VVb, where b and are the scaling exponent and the biomass packing intercept at 1m3 vital volume. Model coefficients for the 50th quantile (median) regression are as follows (95% confidences intervals in parentheses): R2 = 0.86, b = 1.013 (0.988; 1.037) and BP intercept = 0.426 (0.389; 0.471) kg m-3. (DOCX) [file pone.0252080.s002.docx]

**
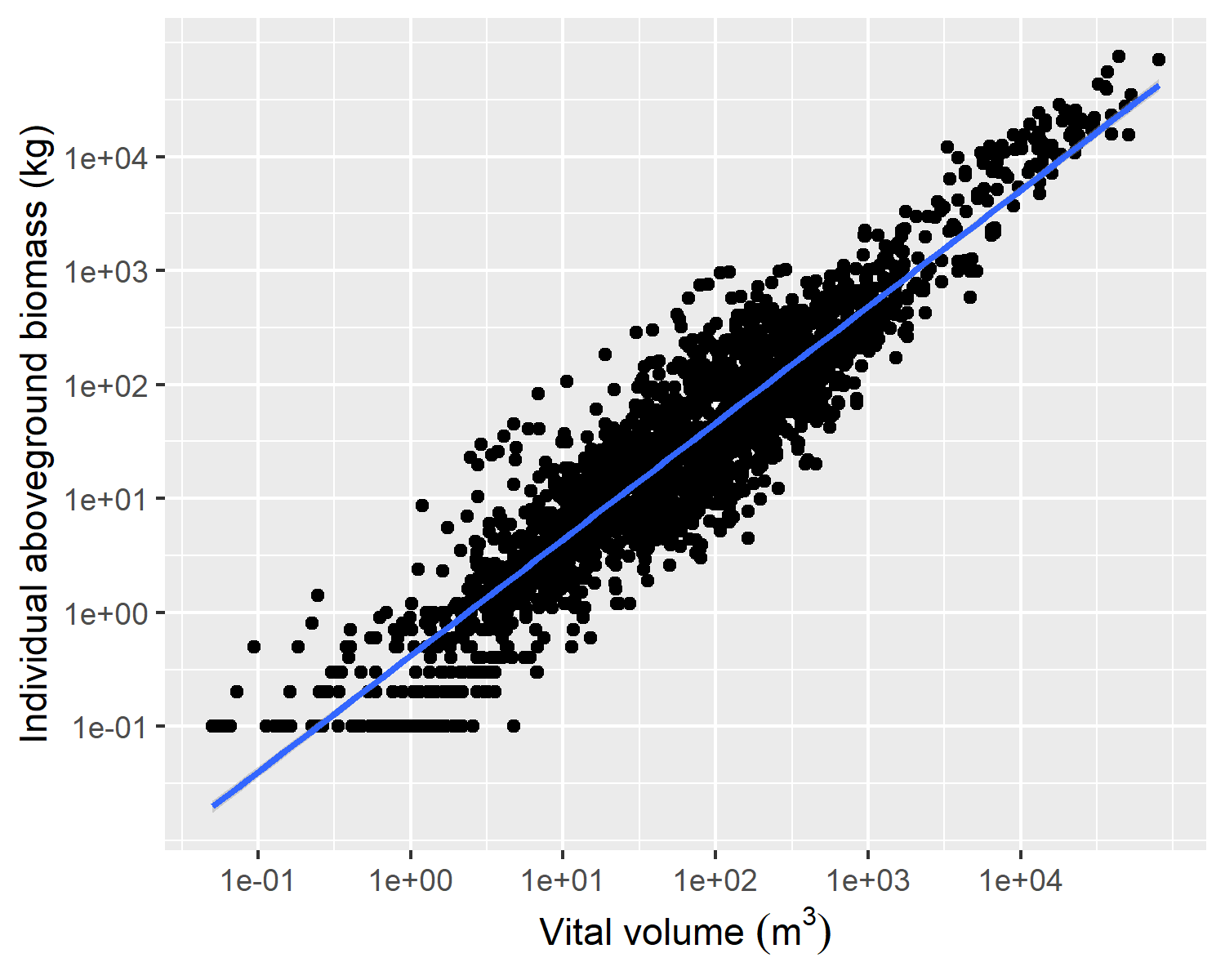
S2 Fig.** Relationship between individual aboveground dry biomass (IAB) and vital volume (VV = CA·H) for 2,395 individual trees across the plant kingdom [19]. The line is the fit of a power function of the form IAB = <BP>·VV^b^, where b and <BP> are the scaling exponent and the biomass packing intercept at 1m^3^ vital volume. Model coefficients for the 50^th^ quantile (median) regression are as follows (95% confidences intervals in parentheses): R^2^ = 0.86, b = 1.013 (0.988; 1.037) and BP intercept = 0.426 (0.389; 0.471) kg m^-3^.
